# Validation of the questionnaire to measure Chilean teachers’ perception of school violence and coexistence management (VI+GEC)

**DOI:** 10.3389/fpsyg.2024.1352399

**Published:** 2024-04-26

**Authors:** Flavio Muñoz-Troncoso, Amy Halberstadt, Isabel Cuadrado-Gordillo, Enrique Riquelme-Mella, Edgardo Miranda-Zapata, Ekaterina Legaz-Vadímisrkaya, Valeria Sepúlveda-Bernales, Claudia Salamanca-Aroca, Gerardo Muñoz-Troncoso

**Affiliations:** ^1^Faculty of Education, Universidad Católica de Temuco, Temuco, Chile; ^2^Facultad de Ciencias Sociales y Artes, Universidad Mayor, Temuco, Chile; ^3^Department of Psychology and Anthropology, Faculty of Education and Psychology, Universidad de Extremadura, Badajoz, Spain; ^4^Department of Psychology, North Carolina State University, Raleigh, NC, United States; ^5^Facultad de Ciencias Sociales y Humanidades, Universidad Autónoma de Chile, Temuco, Chile; ^6^Facultad de Filosofía y Humanidades, Universidad Austral de Chile, Valdivia, Chile; ^7^Faculty of Education, Universidad San Sebastián, Valdivia, Chile

**Keywords:** school violence, management of school coexistence, teacher’s, teacher’s perception, confirmatory factor analysis, structural equation modeling

## Abstract

In this article, we present the development and validation of a psychometric scale that measures the teacher’s perception in the Chilean school system with respect to elements of school violence and coexistence management. The novelty lies in the incorporation of factors that address violence from teachers to students, from students to teachers and coexistence management. A total of 1072 teachers from the Northern, Central, Southern and Metropolitan macro-zones of Chile participated, with ages between 22 and 76 years (M=44.56; SD=10.52) and from 1 to 54 years of work (M=17.14; SD=10.38). 76.3% identify with the female gender and 23.7% with the male gender. Of the teachers, 78.4% worked mainly in the classroom and the rest performed managerial or administrative functions outside the classroom in the school. The school violence and coexistence management questionnaire for teachers (VI+GEC) was used. The validity of the scale was demonstrated by means of Confirmatory Factor Analysis, convergent validity analysis and discriminant validity. Reliability was demonstrated by means of McDonald’s omega coefficient in all the factors of the scale. An analysis with Structural Equation Modeling (SEM) found a mean, and statistically significant influence of the perception of coexistence management on the perception of school violence. The findings are discussed in terms of previous research on school violence and coexistence management.

## 1 Introduction

During the COVID-19 pandemic confinement, studies in several countries reported an increase in domestic violence (Kim and Son, 2023), particularly that perpetrated against children (Cappa and Jijon, 2021), which enabled predicting an increase in violence among students when classes returned (CEPAL, 2020). In Chile, after the reopening of schools, the Ministry of Education (MINEDUC) reported an increase in complaints about problems of school coexistence, most of which were due to violence among students (MINEDUC, 2022). In the same context, students also reported statistically significant differences in school violence, with more violence in face-to-face classes than in the online modality during confinement (Muñoz-Troncoso et al., 2023b). School violence continues to be a problem that affects children and young people, however, research on the subject comes mainly from high-income countries (Kelmendi et al., 2023), which highlights the need to study the phenomenon in developing countries and countries with income disparities. Chile, which has experienced significant economic growth in recent decades but also high income inequality, is a good example, in that its economic growth has not translated into greater well-being for the population (Rojas and Charles-Leija, 2022).

School violence is a serious and complex phenomenon, in which students involved in school violence are affected in many different ways (Meldrum et al., 2022). Victims of school violence may suffer from anxiety, depression, and stress disorders (Gómez-Mármol et al., 2018). Affected students may also decrease in academic performance which is also linked to the risk of dropping out of school (Yang et al., 2021), given the fear and anxiety fostered by the perception of an unsafe school space (Berger, 2019). Both victims and aggressors may present difficulties in socialization, manifested in impulsive and aggressive behaviors (Espelage and Hong, 2019). This set of conditions may be related to the impact on students’ self-esteem and self-confidence, as an impediment to sustaining healthy interpersonal bonds and good academic performance (Lee and Wong, 2022). While it is important to consider the consequences of school violence on the victims, it is also relevant to know the characteristics of the perpetrators. In this regard, one study found that aggressors and aggressor-victims presented relatively low levels of moral judgment and high levels of selfish cognitive distortions (Brugman et al., 2023).

To prevent, reduce, and mitigate the effects of school violence, great value is attributed to the management of coexistence, and there is evidence that intervention programs, as part of that management, can reduce the prevalence of violent events among students (Pina et al., 2021). Several elements are identified as central to the adequate management of coexistence. First and foremost, is the participation of the educational community, which is understood as a collaborative work that includes all the actors of a school, i.e., students, parents, teachers, non-teaching staff and management team (Redon et al., 2023). While recognizing the importance of learning from all actors in the school setting, we focus first on the teachers’ perception of violence for several reasons. In addition to students, teachers are the most prevalent actors in almost all school settings. Although they are not as present as students in every aspect of the school grounds and they cannot be witness to every social interaction, they are trained to scan spaces in which students gather and to recognize multiple forms of violence. They also have a broader historical perspective and interact with many students each day. Perhaps most importantly they are often the first to face conflicts among students and they have the power in the classroom to promote prosocial behaviors (Carbone and Assante Del Leccese, 2023). Altering the interpersonal domain includes the need to promote socioemotional skills that would be helpful in the peaceful resolution of conflicts, thus increasing positive coexistence among students (Nygaard et al., 2023). Teachers have that capacity on a day to day basis, even if administration does not create such curricula standards.

In institutional terms, coexistence management is strengthened when there is clarity regarding the interaction between school members, including positions of total rejection of violence in general. Therefore, strategies for the prevention of violence and well-defined action protocols regarding roles and actions in conflict situations are essential (Aravena et al., 2020). In this sense, it is relevant to work in coordination with other support networks which are mainly in the health and social areas (Medina and Olave, 2022). In this regard, Chilean schools must comply with a series of requirements to ensure adequate management of school coexistence, following guidelines for the development of Internal Regulations, Coexistence Manuals and Protocols for action (MINEDUC, 2018). This is part of the Indicative Performance Standards emanating from the educational policy that governs the school institution (MINEDUC, 2021). From the legal point of view, the above policy is in compliance with Law 20536 on School Violence and Law 20128 Safe Classroom, and in regulatory bodies legislated on school violence and coexistence management in Chile (Muñoz-Troncoso et al., 2023b).

Regarding teachers’ views on school violence at the international level, reports by Han (2021) provided an overview of perceived school violence in Australia, South Korea, the United States of America, and Mexico. The study concludes that in South Korea the overall perception of school violence is higher than in the other participating countries. Teachers in Mexico see violence as a phenomenon more typical of rural schools than urban schools. The case of Australia highlights the increase in violent behavior in recent years. Teachers in the United States see violence as a serious problem that affects mostly urban schools. The study used Likert-type scales as an instrument, which is a particularly efficient method for comparison between countries (Han, 2021). Other international studies have coincided in the high prevalence of school violence, with teachers reporting mostly physical, verbal, and psychological violence (Bourou and Papageorgiou, 2023).

In the Chilean context, several investigations examine the view of teachers regarding the phenomenon of violence in schools, however, few studies incorporate measurement scales. In particular Varela et al. (2021) found that teachers who perceived themselves to be affected by school violence also reported job dissatisfaction. Furthermore, in that study, teachers’ relationship with their schools was affected by various factors related to the school environment, including student victimization, teachers’ perception of the school climate and the level of violence in the environment. Likewise, the research by López et al. (2020) showed a correlation between victimization between teachers and students, evidencing a higher prevalence of verbal violence than physical and sexual violence. Thus, it is highlighted that both physical and verbal victimization between students and teachers represented determining factors in explaining the levels of mutual victimization between them.

Chilean teachers face a critical situation that involves the devaluation of their role, forcing the implementation of various strategies with multiple approaches and possible consequences, a scenario that highlights the work of teachers and their ability to have a significant impact on school coexistence (Carrasco-Aguilar and Luzón, 2019). In addition to the above, in the present research, no psychometric instrument applied in Chile was found that measures teachers’ perception of school violence, among students, between students and teachers, and the management of school coexistence.

There are multiple instruments currently available to explore the views of different actors. From the student perspective, some studies (Guerra et al., 2011) adapt and validate in Chile the Spanish instrument Cuestionario de Violencia Escolar (CUVE) designed by Álvarez et al. (2006), which evaluates violent behaviors in educational establishments from the students’ perspective. There is validation in the Chilean population of the questionnaire that measures students’ perception of peer mistreatment (MIAP) (Lecannelier et al., 2011). The study by Gaete et al. (2021) validated in the Chilean context the Olweus Bully/Victim Questionnaire-Revised Version (OBVQ-R), which measures students’ perception of the forms of bullying. There is also the “Cuestionario de violencia escolar para la No Violencia (CENVI)”, which collects the perception of students on types of violence and the management of school coexistence (Muñoz, 2014; Muñoz et al., 2017; Muñoz-Troncoso et al., 2023a).

In the field of school coexistence, another measurement model is the one that was contributed by Valdés et al. (2018), which consisted of the adaptation and validation in Chile of the School Coexistence Questionnaire designed by Chaparro et al. (2015) to evaluate school coexistence management practices from the perception of students. There is also the instrument developed and validated by Leal-Soto et al. (2022) who suggest the possibility of exploring the management of school coexistence from the students’ perspective through a subscale; however, this would require further study of the psychometric properties of the instrument.

Considering other actors in the school system, Ascorra et al. (2020) designed and validated an instrument that evaluates the management of school coexistence from the perspective of school administrators. The study by López and Valdés (2018) consisted of designing and validating two instruments that evaluate concrete practices of school coexistence management in professionals working in the educational context and in parents and/or mothers. The purpose was to unveil the organizational practices that support the management of school coexistence, in order to contribute to decision-making.

In accordance with Torrego et al. (2022), the present study makes it possible to specify that, although there are instruments that evaluate school coexistence management in Chile, no psychometric instruments were found that jointly evaluate school violence and school coexistence management from the teachers’ perspective.

In view of the above, the general objective is to measure teachers’ perception of school violence and coexistence management by means of the validation of a psychometric scale developed for this purpose. The specific objective is to estimate the impact—from the teachers’ perception—that coexistence management has on school violence and to explore the differences in the perception of violence according to the defined categories. The hypotheses are the following:

*H1*: The proposed four-factor instrument shows adequate goodness-of-fit and reliability indices.

*H2*: There is a statistically significant effect of the coexistence management factor on school violence.

*H3*: There are statistically significant differences between men and women in the perception of violence and school coexistence management.

## 2 Materials and methods

Research with research methodology in psychology and education of the quantitative type, with a descriptive comparative, cross-sectional design (León and Montero, 2015).

### 2.1 Participants

A total of 1072 teachers from the Chilean school system from the North (6.3%), Center (40%), South (23.5%) and Metropolitan (30.2) macro-zones of the country participated. Teachers were between the ages of 22 and 76 years (M=44.56; SD=10.52). and their years of work ranged from 1 to 54 (M=17.14; SD=10.38). Of the participants 76.3% identified with the female gender and 23.7% with the male gender, with 78.4% of them working primarily in the classroom and 21.6% performing other functions in the school. Ten percent reported teaching pre-school (children of 4 and 5 years of age), 55.7% elementary school (children of 6 to 13 years of age) and 34.3% middle school (young people from 14 to 17 years old). Of the teachers, 51% belonged to municipal schools, 34.8% to private subsidized schools and 14.2% to private schools; 76.3% identified as female and 23.7% as male.

All of them participated voluntarily through a letter of informed consent, and a non-probabilistic sampling was carried out by accessibility in the indicated macro-areas.

### 2.2 Instrument

The 21-item school violence and coexistence management questionnaire for teachers (VI+GEC) was applied. It is a Likert-type scale with four scales. Three of the four scales were adapted from the CENVI questionnaire for students (Muñoz-Troncoso et al., 2023a), in order to create a measure that allowed for comparability across students and teachers. Factor 1, called Violence among students (VEE), is composed of five items. Factor 2, called Violence from teacher to student (VPE), is composed of six items. The new Factor 3, called Student-to-teacher violence (STV), is composed of four items. Factor 4, called Management of School Coexistence (GCE), is composed of six items. The indicators of factors 1, 2 and 4 were adapted from the CENVI questionnaire (for students) of Muñoz-Troncoso et al. (2023a), the indicators of factor 3 were elaborated for the present study. For each item, teachers were asked to respond on a scale in which 1=Never and 6=Always. Thus, for the three violence subscales, the higher the score the greater the violence, and for coexistence management, the higher the score the higher the evaluation of management.

### 2.3 Procedure

The study is nested in the FONDECYT Regular 1191956 project “Family and school education: Emotional socialization in contexts of social and cultural diversity,” and reviewed and approved by the Research Ethics Committee of the Universidad Católica de Temuco (Chile). The instrument is hosted on a web platform, which begins with a description of the questions, an informed consent and confidentiality notice with details about the characteristics of the research, the instrument, and the time required to respond. The voluntary nature of participation was made explicit, guaranteeing anonymity and data protection. The study was conducted according to the international deontological guidelines referred to in the Declaration of Helsinki and the Singapore Declaration, as well as those referred to in Chile by Law 20120.

### 2.4 Plan for analysis

The adaptation and creation of items, in addition to the proposed structure, considered content validity by means of inter-judgment of experts. The normality of the indicators was evaluated using the Kolmogorov–Smirnov test to choose the subsequent analyses. Based from previous reports from students and teachers, we hoped that the data would reveal a skewed distribution reflective of lower rates of violence. Thus, we anticipated that our next step, specifically, a Confirmatory Factor Analysis (CFA) would be carried out, as appropriate for non-normative data, using the Maximum Likelihood adjusted by Mean and Variance (MLMV). The Chi-square statistic would be optimal if the ratio with its degrees of freedom is less than 3:1. The goodness-of-fit indices considered are the root mean squared error of approximation (RMSEA), expecting values less than 0.5 as excellent or less than 0.7 as acceptable. The comparative fit index (CFI) and the Tucker-Lewis index (TLI) would be excellent with values greater than 0.95 and acceptable with values greater than 0.9.

Convergent validity was assessed where each factor must present: (1) standardized loadings with values greater than 0.5 and statistical significance level *p*-value less than 0.05; (2) average variance extracted (AVE) with values greater than 0.5; and (3) composite reliability with values greater than 0.7. The discriminant validity assessment consisted of comparing the AVE with the shared variance, where the AVE of a factor should be greater than the square of the correction between it and the other factors. The reliability of the measurement model was evaluated through McDonald’s omega coefficient, considering values greater than 0.65 as admissible, greater than 0.7 as acceptable, between 0.8 and 0.9 as good, and above 0.9 as excellent.

An analysis was carried out through structural equation modeling (SEM) to measure the effect of coexistence management on school violence, proposing that factors 1 to 3 can be measured by a second-order factor (G1) and thus assess the effect that factor 4 (coexistence management) has on it.

Subsequently, we calculated scales using k-means cluster analysis, determining three clusters to differentiate high, medium, and low levels in each factor. Finally, teachers were grouped according to their level of perception of each factor.

The last stage consisted of reviewing the measurement invariance of the questionnaire for all the defined categories. The configural invariance is achieved by fulfilling the criteria of a CFA, and the metric invariance is achieved if the variations of CFI and RMSEA between it and the configural invariance are not significant. Similarly, scalar invariance is evidenced if the CFI and RMSEA variations between it and the metric invariance are not significant. In this regard, it is expected: ΔCFI <0.01, ΔRMSEA<0.015. Given the above finding, the differences between pairs of groups of the defined categories were checked through the Mann–Whitney U-test.

Data were analyzed with Microsoft Excel v.16.74 (Microsoft, 2023), SPSS v.23 (IBM Corp, 2020), JASP v.0.17.21 (JASP Team, 2023), RStudio v. 2023.06.0 + 421 (RStudio Team, 2022), and G*Power v. 3.1.9.6 (Buchner et al., 2020).

## 3 Results

Content validity made it possible to retain the proposed four-factor model shown in Table 1.

**Table 1:** Structure of the measurement model.

Factor	Name	Abbreviation	Items	Variables
F1	Student-to-student violence	SSV	5	x1–x5
F2	Teacher-to-student violence	TSV	6	x6–x11
F3	Violence from student to teacher	VST	4	x12–x15
F4	School coexistence management	MSC	6	x16–x21

Source: Prepared by the authors.

**Figure 1:**
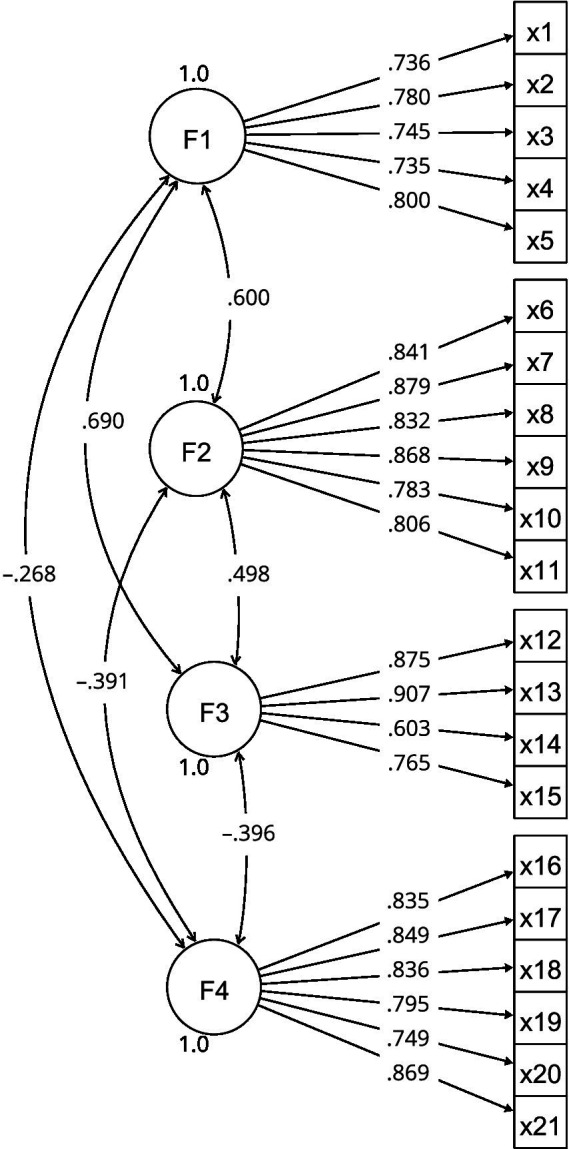
Measurement model diagram. Source: Prepared by the authors.

The saturations and correlations are shown in Figure 1.

The Kolmogorov–Smirnov test showed that the data did not resemble a normal distribution (*p*-value<0.001). The AFC presented X^2^ = 643.904; DF = 183 and *p* < 0.001. The model is a good fit for the data (RMSEA = 0.048; CFI = 0.967; and TLI = 0.962).

The convergent validity of the model is supported in that there are saturations greater than 0.5, AVE greater than 0.5, and composite reliability greater than 0.7 (Table 2). Discriminant validity is evidenced in that the AVE of each factor is greater than the squared correlation between factors (Table 3). The scale has good reliability given that all factors reach ω = 0.9 (Table 2).

**Table 2:** Convergent, discriminant, and reliability validity indicators.

						Factors			
Factors		Saturations				F1	F2	F3	F4
	Abbreviation	Minimum	Maximum	Reliability	AVE	SSV	TSV	VST	MSC
F1	SSV	0.736	0.801	0.9	0.577		0.360	0.476	0.072
F2	TSV	0.841	0.806	0.9	0.698	0.600		0.239	0.153
F3	VST	0.876	0.765	0.9	0.635	0.690	0.489		0.157
F4	MSC	0.836	0.869	0.9	0.678	−0.268	−0.391	−0.396	

SSV= Student-Student Violence. TSV= Teacher-Student Violence. VST= Violence from student to teacher. MSC= Management of school coexistence. Correlations between factors are shown below the diagonal. The squares of the correlations between factors are shown above the diagonal. Source: Prepared by the authors.

**Table 3:** Levels according to cutoff points for each factor.

Factor	Abbreviation	Low	Medium	High
F1	SSV	5–11	12–17	18–30
F2	TSV	6–12	13–20	21–36
F3	VST	4–8	9–14	15–24
F4	MSC	6–18	19–27	28–36

Source: Prepared by the authors.

**Figure 2:**
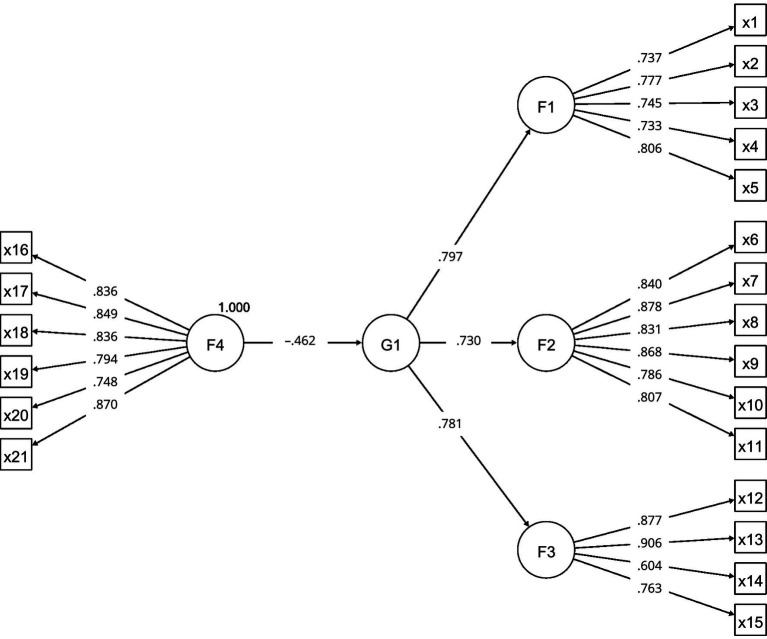
SEM path diagram. Source: Prepared by the authors.

The model proposed to measure the direct effect of coexistence management on school violence (Figure 2) presents a good fit to the data (RMSEA = 0.058; CFI = 0.951; TLI = 0.945). The effect of Coexistence Management on School Violence is of medium magnitude, statistically significant (γ = −0.462; *p* < 0.001), and shows a good confidence interval (range = −0.462; L = −0.537; U = −0.387).

The cutoff points for the factors are presented in Table 3. For factors 1 to 3, the higher the score, the higher the perception of violence; for factor 4, the higher the score, the better the evaluation of coexistence management.

A good fit of the model to the data was found in all groups of the categories analyzed. Except for the macrozone grouping, scalar invariance was achieved in the categories reviewed (ΔCF1 < 0.01; ΔRMSEA<0.015). The differences between groups are presented in Table 4.

**Table 4:** Comparisons between pairs of groups using the Mann–Whitney U-test.

Factor	Group 1	Mdn.	Rng.	Group 2	Mdn.	Rng.	*p*	*U*	*1-β*	*d*
SSV	Female	13	25	Male	13	21	0.042	95965.5	0.39	0.01
	Classroom	13	24	Administrative	13	25	0.619	95373.0	0.63	0.02
	Pre-school	9	19	Primary	13	24	<0.001	15506.5	1.00	0.90
	Pre-school	9	19	Secondary	14	25	<0.001	7613.5	1.00	0.90
	Primary	13	24	Secondary	14	25	0.002	96947.0	0.28	0.17
	Municipal	13	25	Subsidized	12	20	<0.001	88281.5	0.87	0.24
	Municipal	13	25	Private	13	22	0.187	38675.0	0.51	0.12
	Subsidized	12	20	Private	13	22	0.199	26330.0	0.87	0.12
	Urban	13	25	Rural	11	20	<0.001	47694.5	0.97	0.45
TSV	Female	11	30	Male	11	21	0.127	98140.5	0.38	0.09
	Classroom	11	30	Administrative	11	26	0.107	90730.0	0.84	0.11
	Pre-school	8	26	Primary	11	30	<0.001	23271.5	0.50	0.34
	Pre-school	8	26	Secondary	12	26	<0.001	12037.5	1.00	0.79
	Primary	11	30	Secondary	12	26	0.003	97349.0	0.20	0.15
	Municipal	11	30	Subsidized	10	26	0.001	89332.5	0.59	0.19
	Municipal	11	30	Private	12	21	0.189	38687.0	0.38	0.10
	Subsidized	10	26	Private	12	21	<0.001	22979.0	0.39	0.30
	Urban	11	30	Rural	9	23	0.002	55859.5	0.25	0.22
VST	Female	7	20	Male	7	14	0.845	103890.0	0.89	0.07
	Classroom	7	20	Administrative	6	14	<0.001	80586.0	0,85	0.33
	Pre-school	7	17	Primary	7	20	0.426	30409.5	0.56	0.09
	Pre-school	7	20	Secondary	7	14	0.191	18064.0	0.32	0.09
	Primary	7	20	Secondary	7	14	0.331	105790.0	0.33	0.01
	Municipal	8	20	Subsidized	7	15	<0.001	87950.0	0.92	0.26
	Municipal	8	20	Private	6	15	<0.001	31165.0	0.84	0.41
	Subsidized	7	15	Private	6	15	0.025	24857.5	0.29	0.17
	Urban	7	20	Rural	6	11	<0.001	48260.5	0.95	0.45
MSC	Female	26	30	Male	27	29	0.012	93926.0	0.59	0.01
	Classroom	25	30	Administrative	30	30	<0.001	66589.0	1.00	0.59
	Pre-school	24	25	Primary	26	30	0.019	27415.0	0.47	0.24
	Pre-school	24	25	Secondary	26	30	0.142	17,854	0.55	0.16
	Primary	26	30	Secondary	26	30	0.123	103365.0	0.43	0.09
	Municipal	25	30	Subsidized	26	30	0.048	94189.5	0.63	0.13
	Municipal	25	30	Private	27	30	0.712	40758.5	0.73	0.04
	Subsidized	26	30	Private	27	30	0.290	26682.0	0.47	0.09
	Urban	26	30	Rural	28	30	0.166	61652.5	0.49	0.11

Source: Prepared by the authors.

## 4 Discussion and conclusion

Based on the obtained results, it is possible to argue that the VI + GEC questionnaire is a valid and reliable instrument that measures teachers’ perceptions of school violence and coexistence management. Having a psychometrically developed questionnaire such as the one presented is relevant for understanding the phenomenon of violence in schools and for the analysis of coexistence management. A comprehensive perspective in this regard can favor decision-making (Torrego et al., 2022) to prevent and mitigate the effects of violence (Muñoz-Troncoso et al., 2023b). Thus, the novelty of the study is the development of a psychometric scale—for teachers—that incorporates factors of mutual violence between students and teachers, in addition to the factor of school coexistence. This scale also made it possible to measure the effect of coexistence management on school violence.

Regarding the psychometric properties of the designed instrument, the content validity allowed maintaining the proposed model of four factors that refer to (1) violence between students; (2) teacher-to-student violence; (3) student-to-teacher violence; and (4) school coexistence management. This structure is consistent with the National Policy on School Coexistence (MINEDUC, 2019). Convergent validity was demonstrated, indicating that each factor of the questionnaire is significantly related to its respective construct and in the ways we predicted. Likewise, discriminant validity was evidenced, given that there is no high correlation between the factors that comprise it. The confirmatory factor analysis identified a good fit of the proposed model to the data, showing that the indicators measure the latent variables to which they conform. Similarly, all the factors of the instrument showed good reliability indicators. In addition, scalar invariance was achieved in the defined categories (except Macrozone), which allowed for an unbiased review of the differences between groups.

A relevant finding is that, from the obtained results, it was evident that there is a direct effect of the perception of coexistence management on the perception of school violence since a medium and statistically significant effect was found. This finding is in agreement with what was stated by Pina et al. (2021) regarding the importance of coexistence management with respect to school violence and the need to generate efficient strategies in the face of conflict situations (Aravena et al., 2020). It also corroborates the importance of training tools that facilitate the management of coexistence and the approach to situations of violence (Morales et al., 2014). It is important to note that this result is opposite to that reported by Muñoz-Troncoso et al. (2023b), where a null effect of management on violence was found. However, this effect can be explained by the fact that the questionnaire used in that study measures the perception of students, actors who, according to López et al. (2023), have little or no impact on coexistence management.

The final analysis showed groups with statistically significant differences. *Gender*: In the VST factor, women perceived more violence than men, and in the MSC factor, women gave better ratings than men. This difference could be due to the fact that women present better moral judgment and less egoistic cognitive distortions than men (Brugman et al., 2023), so they make a better assessment of situations, identifying facts of violence that might not be evidenced by men. *Function*: In the VST dimension, classroom teachers see more violence, and in the MSC dimension, ‘administrative’ teachers value the factor better than teachers who work in the classroom. This finding can be explained by the fact that teachers exposed to situations of violence may see the coherence with which they perceive their school community deteriorate (Morales et al., 2014). *School level*: In the SSV and TSV factors, elementary school teachers see more violence than pre-school teachers, and secondary school teachers see more violence than pre-school and elementary school teachers. In the MSC dimension, elementary school teachers give a better evaluation than pre-school teachers. A better evaluation could occur because supervision decreases as the age of students increases, and many acts of violence occur in situations where supervision is less strict (Bourou and Papageorgiou, 2023).

*Type of school*: In the SSV dimension, teachers in municipal schools see more violence than those in private subsidized schools; in TSV, teachers in municipal schools see more violence than those in private subsidized schools, and teachers in private schools see more violence than those in private subsidized schools. For the VST dimension, teachers in private subsidized schools perceived more violence than those in private schools, and teachers in municipal schools perceived more violence than those in private subsidized schools and those in private schools. In MSC, teachers in private subsidized schools perceived better management than those in municipal schools. This finding could be related to the concentration of students at low socioeconomic levels in Chilean municipal schools. Children from this group are more exposed to community violence, which impacts their relationship with the teacher and the school and can negatively affect the school environment, which in turn influences the wellbeing of students and teachers (Varela et al., 2021). Similarly, in municipal schools, economic, infrastructure, and management resources are lower than in private subsidized schools, with private schools having more resources (Guerra et al., 2011). *Location*: In the three types of violence measured, teachers in urban schools saw more violence than those in rural schools. This finding is related to the existing perception of rural education as a space with a lower risk of violence dynamics, which, in the perspective of Núñez et al. (2023), has been reinforced by the School Inclusion Law and has even led to an increase in enrollment in these schools from students living in urban centers.

Being able to assess the perception of teachers is of great relevance, because it influences the quality of teaching and learning processes (Torrego et al., 2022), job dissatisfaction, and the relationship that teachers have with their schools (Varela et al., 2021). This fact confirms the primordial role of teachers’ work in the promotion and implementation of actions in school coexistence (Carrasco-Aguilar and Luzón, 2019). In addition, the teacher’s perception is particularly relevant, given that it differs from what students perceive (Ascorra and López, 2019).

It is possible to argue that, to obtain a comprehensive measurement of school violence and coexistence management, the joint application of the VI + GEC (developed in the present study) and CENVI questionnaires (Muñoz-Troncoso et al., 2023a) is necessary. The former allows us to know the perception of the teaching staff, and the latter allows us to approach the students’ perspectives and experiences. This finding could contribute to a general appreciation of the different actors in the educational communities (D’auria-Tardeli et al., 2023), considering the importance of developing evaluation and monitoring systems that contribute to the implementation of management models by school directors and managers (Ascorra et al., 2021).

The present study fulfills the proposed objective since the perception of Chilean teachers regarding school violence and coexistence management was measured, which implied the validation of a psychometric scale elaborated—ipso facto—for this purpose. Regarding the hypotheses raised, it can be pointed out:

“H1: The proposed instrument evidence adequate goodness-of-fit and reliability indexes.” It is confirmed that, since the confirmatory factor analysis showed a good fit of the proposed model to the data, convergent and discriminant validity was evidenced, along with demonstrating good reliability indicators for all the factors of the scale.

“H2: There is a statistically significant effect of the coexistence management factor on school violence”. It is confirmed due to the evidence of a direct, medium, and statistically significant effect of the perception of coexistence management on the perception of school violence.

“H3: There are statistically significant differences between men and women regarding the perception of violence and management of school coexistence”. It is confirmed by evidence that women perceive more violence among students than men. Similarly, women perceive a better management of school coexistence than men.

Limitations. Despite the large sample size, we note that the sample is non-probabilistic due to accessibility, and turned out not to be proportional among the defined macro-zones nor representative of the teaching staff of the Chilean population, which prevents the generalization of the results. Although the proposed cutoff points arise from the sample accessed, it is feasible that the instrument becomes an applicable tool in Chilean educational establishments to evaluate school violence and the management of coexistence from the teachers’ perspective. Certainly, the measure can be used to assess chronological shifts within schools or regions to assess success of programs designed to reduce violence in schools. Its implementation could contribute to understanding the specific panorama of the factors measured by the VI + GEC, allowing informed and preventive decision-making in the face of school violence, with pertinent actions from the management of coexistence in school spaces.

## Data availability statement

The raw data supporting the conclusion of this article will be made available by the authors, without undue reservation.

## Ethics statement

The studies involving humans were approved by Research Ethics Committee of the Universidad Católica de Temuco. The studies were conducted in accordance with the local legislation and institutional requirements. The participants provided their written informed consent to participate in this study.

## Author contributions

FM-T: Writing – review & editing, Writing – original draft, Visualization, Validation, Software¸ Resources, Methodology, Investigation¸ Formal analysis, Data curation, Conceptualization. AH: Writing – review & editing, Supervision, Resources, Conceptualization. IC-G: Writing – review & editing, Supervision, Resources, Investigation, Conceptualization. ER-M: Writing – review & editing, Writing – original draft, Supervision, Resources, Project administration, Methodology, Funding acquisition, Conceptualization. EM-Z: Writing – review & editing, Validation, Supervision, Software, Resources, Methodology. EL-V: Conceptualization, Writing – review & editing, Writing – original draft, Resources. VS-B: Conceptualization, Writing – review & editing, Writing – original draft, Resources, Investigation. CS-A: Conceptualization, Writing – review & editing, Writing – original draft, Investigation. GM-T: Writing – review & editing, Writing – original draft, Supervision, Resources, Conceptualization.

## References

[ref1] Álvarez L. Álvarez-García D. González-Castro P. Núñez J. C. González-Pineda J. A. (2006). Evaluación de los comportamientos violentos en los centros educativos. Psicothema 18, 686–695. 17296104

[ref2] Aravena F. Ramirez J. Escare K. (2020). Acciones en convivencia escolar de equipos directivos y líderes escolares en Chile: ¿Qué? ¿Con quiénes? y ¿Dónde? Perspect. Educ. 59, 45–65. doi: 10.4151/07189729-Vol.59-Iss.2-Art.1045

[ref3] Ascorra P. Cárdenas K. Álvarez F. (2020). Gestión de la convivencia escolar a nivel intermedio: Diseño y validación de una escala. Rev. Evaluar 20, 1–19. doi: 10.35670/1667-4545.v20.n3.31700

[ref4] Ascorra P. Cárdenas K. Torres J. (2021). Niveles de Progresión de Gestión de la Convivencia Escolar a Nivel Intermedio en Chile. Rev. Int. Educ. Para Justicia Soc. 10, 227–243. doi: 10.15366/riejs2021.10.1.014

[ref5] Ascorra P. López V. (2019). Una década de investigación en convivencia escolar Ediciones Universidad de Valparaíso. Available at: http://librosonline.ucv.cl/index.php/pucv/catalog/view/5/16/52-1

[ref6] Berger E. (2019). Multi-tiered approaches to trauma-informed care in schools: a systematic review. Sch. Ment. Heal. 11, 650–664. doi: 10.1007/s12310-019-09326-0

[ref8] Bourou A. Papageorgiou E. (2023). Prevalence of aggressive behavior in Greek elementary school settings from teachers’ perspectives. Behav. Sci. 13, 1–14. doi: 10.3390/bs13050390, PMID: 37232627 PMC10215869

[ref9] Brugman D. Van Der Meulen K. Gibbs J. C. (2023). Moral judgment, self-serving cognitive distortions, and peer bullying among secondary school adolescents. J. Moral Educ. 1–21, 1–21. doi: 10.1080/03057240.2023.2209289

[ref10] Buchner A. Erdfelder E. Faul F. Lang A.-G. (2020). *G*Power* (3.1.9.6) [Computer software]. Heinrich-Heine-Universität. Available at: https://www.psychologie.hhu.de/arbeitsgruppen/allgemeine-psychologie-und-arbeitspsychologie/gpower

[ref11] Cappa C. Jijon I. (2021). COVID-19 and violence against children: a review of early studies. Child Abuse Negl. 116:105053. doi: 10.1016/j.chiabu.2021.105053, PMID: 33965215 PMC9754317

[ref12] Carbone A. Assante Del Leccese R. (2023). The methodology of psychological clinical intervention in school settings: case studies with students with special emotional and educational needs. Educ. Sci. 13:463. doi: 10.3390/educsci13050463

[ref13] Carrasco-Aguilar C. Luzón A. (2019). Respeto docente y convivencia escolar: Significados y estrategias en escuelas chilenas. Psicoperspectivas. Individuo y Sociedad 18, 1–11. doi: 10.5027/psicoperspectivas-Vol18-Issue1-fulltext-1494

[ref14] CEPAL. (2020). *La educación en tiempos de la pandemia de COVID-19*. CEPAL. Available at: https://repositorio.cepal.org/handle/11362/45904

[ref15] Chaparro A. Caso J. Fierro M. C. Díaz C. (2015). Desarrollo de un instrumento de evaluación basado en indicadores de convivencia escolar democrática, inclusiva y pacífica. Perfiles Educativos 37, 20–41.

[ref16] D’auria-Tardeli D. Barros L. D. S. Tessaro M. Alves V. T. (2023). Percepções de professores sobre clima educacional na educação infantil de São Bernardo do Campo. Educ. Pesqui. 49:e249251. doi: 10.1590/s1678-4634202349249251por

[ref17] Espelage D. L. Hong J. S. (2019). “School climate, bullying, and school violence” in School safety and violence prevention: Science, practice, policy. eds. Mayer M. J. Jimerson S. R. (American Psychological Association), 45–69. doi: 10.1037/0000106-003

[ref18] Gaete J. Valenzuela D. Godoy M. I. Rojas-Barahona C. A. Salmivalli C. Araya R. (2021). Validation of the revised Olweus bully/victim questionnaire (OBVQ-R) among adolescents in Chile. Front. Psychol. 12:578661. doi: 10.3389/fpsyg.2021.578661, PMID: 33912096 PMC8072054

[ref19] Gómez-Mármol A. Sánchez-Alcaraz Martínez B. J. Valero-Valenzuela A. Cruz-Sánchez E. D. L. (2018). Perceived violence, sociomoral attitudes and behaviours in school contexts. J. Hum. Sport Exerc. 13, 138–148. doi: 10.14198/jhse.2018.131.14

[ref20] Guerra C. Álvarez-García D. Dobarro A. Núñez J. C. Castro L. Vargas J. (2011). Violencia escolar en estudiantes de educación secundaria de Valparaíso (Chile). Rev. Iberoam. Psicol. Salud 2, 75–98.

[ref21] Han S. (2021). School violence in South Korea: international comparative analysis. Springer, Singapore

[ref22] IBM Corp. (2020). *SPSS statistics* (27.0.1.0) [Computer software]. IBM Corp. Available at: https://www.ibm.com/cl-es/analytics/spss-statistics-software

[ref23] JASP Team. (2023). *JASP* (0.17.21 (Apple Sollicon)) [Computer software]. University of Amsterdam. Available at: https://jasp-stats.org/download/

[ref24] Kelmendi K. Arënliu A. Benbenishty R. Astor R. Hyseni Z. Konjufca J. (2023). An exploratory study of secondary school student victimization in Kosovo and its correlates. J. Sch. Violence 22, 459–473. doi: 10.1080/15388220.2023.2214736 PMC12493020 41048516

[ref7] Kim B. Son K-B. (2023). Tightened social distancing measures and increased violence during the COVID-19 pandemic in South Korea. Front. Psychol. 14:1152693. doi: 10.3389/fpsyg.2023.1152693, PMID: 37469889 PMC10352582

[ref25] Leal-Soto F. Cuadros O. Ortiz-Iñíguez N. Zenteno-Osorio S. (2022). Desarrollo y Evidencia de Validez de Constructo de un Instrumento para Evaluar Experiencia Escolar. Rev. Iberoam. Diagn. Ev. Aval. Psicol. 63, 147–162. doi: 10.21865/RIDEP63.2.11

[ref26] Lecannelier F. Varela J. Rodríguez J. Hoffmann M. Flores F. Ascanio L. (2011). Validación del Cuestionario de Maltrato entre Iguales por Abuso de Poder (MIAP) para escolares. Rev. Med. Chile 139, 474–479. doi: 10.4067/S0034-98872011000400009, PMID: 21879186

[ref27] Lee C. Wong J. S. (2022). Examining the effects of teen dating violence prevention programs: a systematic review and meta-analysis. J. Exp. Criminol. 18, 1–40. doi: 10.1007/s11292-020-09442-x

[ref28] León O. Montero I. (2015). Metodos de Investigacion Psicologia y Educacion: Las Tradiciones Cuantitativa y Cualitativa. 4th Edn. Madrid: McGraw-Hill.

[ref29] López V. Benbenishty R. Astor R. A. Ascorra P. González L. (2020). Teachers victimizing students: contributions of student-to-teacher victimization, peer victimization, school safety, and school climate in Chile. Am. J. Orthopsychiatry 90, 432–444. doi: 10.1037/ort0000445, PMID: 32134312

[ref30] López V. Valdés R. (2018). Construcción y validación de instrumentos para evaluar prácticas de convivencia escolar en profesionales y padres. Actual. Investig. Educ. 18, 1–29. doi: 10.15517/aie.v18i3.34328

[ref31] López V. Valdés R. Valle L. R.-C. D. Baleriola E. (2023). Traducciones heterogéneas de la(s) política(s) de convivencia escolar en Chile. Rev. Bras. Educ. 28, 1–26. doi: 10.1590/s1413-24782023280058

[ref32] Medina B. Olave M. (2022). Significados que atribuyen los encargados de convivencia escolar a las conductas violentas presentes en estudiantes agresores de establecimientos educacionales en Temuco. Medisur 20:2022. Available at: https://medisur.sld.cu/index.php/medisur/article/view/5346

[ref33] Meldrum R. Patchin J. Young J. Hinduja S. (2022). Bullying victimization, negative emotions, and digital self-harm: testing a theoretical model of indirect effects. Deviant Behav. 43, 303–321. doi: 10.1080/01639625.2020.1833380

[ref34] Microsoft. (2023). *Microsoft Excel* (16.74 (23061100)) [Computer software]. Microsoft. Available at: https://www.microsoft.com/

[ref35] MINEDUC. (2018). *Circular que imparte instrucciones sobre reglamentos internos de los establecimientos educacionales de enseñanza básica y media con reconocimiento oficial del Estado* [Superintendencia de Educación]. Available at: https://www.supereduc.cl/contenidos-de-interes/nueva-circular-normativa-reglamento-interno/

[ref36] MINEDUC. (2019). *Política Nacional de Convivencia Escolar*. División de Educación General. MINEDUC. Available at: https://bibliotecadigital.mineduc.cl/handle/20.500.12365/4472

[ref37] MINEDUC. (2021). *Estándares indicativos de desempeño*. Unidad de Currículum y Evaluación. MINEDUC. Available at: https://bibliotecadigital.mineduc.cl/handle/20.500.12365/14361

[ref38] MINEDUC. (2022). *Cuenta pública participativa 2021*. Super Intendencia de Educación. Available at: https://www.supereduc.cl/wp-content/uploads/2022/05/cuenta_2021.pdf

[ref39] Morales M. López V. Bilbao M. Á. Villalobos B. Oyarzún D. Olavarría D.. (2014). El papel mediador de la capacitación docente en el manejo de la violencia escolar sobre el bienestar social de profesores. Ter. Psicol. 32, 217–226. doi: 10.4067/S0718-48082014000300004

[ref40] Muñoz F. (2014). *Elaboración y Validación Psicométrica del Cuestionario de Convivencia Escolar Para la No Violencia (CENVI), en Escolares de la Ciudad de Temuco, Chile* [Tésis magíster, Universidad Católica de Temuco]. Available at: http://repositoriodigital.uct.cl/handle/10925/2453

[ref41] Muñoz F. Becerra S. Riquelme E. (2017). Elaboración y validación psicométrica del cuestionario de convivencia escolar para la no violencia (CENVI). Estud. Pedagóg. (Valdivia) 43, 205–223. doi: 10.4067/S0718-07052017000300012

[ref42] Muñoz-Troncoso F. Cuadrado-Gordillo I. Riquelme-Mella E. Miranda-Zapata E. Ortiz-Velosa E. (2023a). Perception of school violence: indicators of normalization in Mapuche and non-Mapuche students. Int. J. Environ. Res. Public Health 20, 1–19. doi: 10.3390/ijerph20010024, PMID: 36612344 PMC9819544

[ref43] Muñoz-Troncoso F. Cuadrado-Gordillo I. Riquelme-Mella E. Muñoz-Troncoso G. Miranda-Zapata E. Bizama-Colihuinca K.. (2023b). Validation of an abbreviated scale of the CENVI questionnaire to evaluate the perception of school violence and coexistence management of Chilean students: differences between pandemic and post-pandemic. Behav. Sci. 13:686. doi: 10.3390/bs13080686, PMID: 37622826 PMC10451708

[ref9001] Núñez C. G. Díaz M. González B. (2023). ¿Elección de escuela o peregrinaje escolar?: Trayectorias de estudiantes que migran de escuelas urbanas a rurales en Chile. Revista Complutense de Educación, 34, 265–275. doi: 10.5209/rced.77343

[ref44] Nygaard M. A. Ormiston H. E. Heck O. C. Apgar S. Wood M. (2023). Educator perspectives on mental health supports at the primary level. Early Childhood Educ. J. 51, 851–861. doi: 10.1007/s10643-022-01346-x, PMID: 35528139 PMC9062637

[ref45] Pina D. Ruiz-Hernández J. A. Llor-Esteban B. Matás-Castillo M. Pagán-Escribano M. Puente-López E. (2021). “Count on me” program to improve school coexisting in primary education. Child Youth Serv. Rev. 127:106121. doi: 10.1016/j.childyouth.2021.106121

[ref46] Redon S. Vallejos N. Belaustegui C. (2023). Nebulosa de lo público en una escuela chilena: Un estudio de caso. Educ. Policy Anal. Arch. 31, 1–24. doi: 10.14507/epaa.31.7558

[ref47] Rojas M. Charles-Leija H. (2022). Chile, milagro de crecimiento económico, pero… ¿y el bienestar? Perf. Latinoam. 30, 1–28. doi: 10.18504/pl3059-005-2022

[ref48] RStudio Team. (2022). *RStudio: Integrated Development for R. RStudio* (2022.07.1 Build 554) [Computer software]. PBC. Available at: http://www.rstudio.com/

[ref49] Torrego J. C. Gomariz M. Á. Caballero P. Monge C. (2022). Cuestionario de convivencia escolar desde un modelo integrado para profesores. Aula Abierta 51, 93–104. doi: 10.17811/rifie.51.1.2022.93-104

[ref50] Valdés R. López V. Chaparro A. (2018). Convivencia escolar: Adaptación y validación de un instrumento mexicano en Chile. Rev. Electron. Investig. Educ. 20, 80–91. doi: 10.24320/redie.2018.20.3.1720

[ref51] Varela J. J. Melipillán R. González C. Letelier P. Massis M. C. Wash N. (2021). Community and school violence as significant risk factors for school climate and bonding of teachers in Chile: a national hierarchical multilevel analysis. J. Community Psychol. 49, 152–165. doi: 10.1002/jcop.22470, PMID: 33190282

[ref52] Yang Y. Qin L. Ning L. (2021). School violence and teacher professional engagement: a cross-National Study. Front. Psychol. 12:628809. doi: 10.3389/fpsyg.2021.628809, PMID: 33935880 PMC8082018

