# Supplementary material for: Validation of the questionnaire to measure Chilean teachers’ perception of school violence and coexistence management (VI+GEC)
Source: Front Psychol. 2024 Apr 26;15:1352399. doi: 10.3389/fpsyg.2024.1352399 (PMC11085254; doi:10.3389/fpsyg.2024.1352399)
Supplement: Supplementary file 1 [file Data_Sheet_1.ZIP › scales_items.pdf]

Questionnaire to measure Chilean Teachers' Perception of School Violence and Coexistence Management (VI+GEC). *[flaviomunoz@gmail.com ; eriquelme@uct.cl]*

Paper	BBDD	Ítem
X1	VEE01	Hay estudiantes que se burlan y/o insultan a sus compañeros/as.
X2	VEE05	Algunos estudiantes rompen y/o roban pertenencias de sus compañeros.
X3	VEE06	Hay estudiantes que no dejan entrar en su grupo a algunos/as compañeros/as.
X4	VEE09	Algunos/as estudiantes agreden 'anónimamente' a otros/as, a través de redes sociales y/o dispositivos digitales de comunicación.
X5	VEE10	Hay estudiantes que intencionan contacto físico de connotación sexual no solicitado, hacia otros/as estudiantes.
X6	VPE01	Hay profesores/as que le tienen 'mala barra' a algunos estudiantes.
X7	VPE02	En el colegio, hay profesores/as que se burlan y/o ridiculizan a algunos estudiantes.
X8	VPE03	Hay profesores/as que hacen sus clases sin escuchar a la mayoría de los estudiantes y/o ignorándolos.
X9	VPE04	En el colegio, hay profesores/as que hacen comentarios que ofenden y/o intimidan a algunos estudiantes.
X10	VPE05	Algunos/as profesores/as llaman la atención a los estudiantes con gritos y/o golpes en la mesa.
X11	VPE07	Algunos/as profesores/as intencionan malas notas hacia ciertos estudiantes.
X12	VEP01	En este colegio, hay estudiantes que gritan y/o insultan a los/as profesores/as.
X13	VEP02	Algunos estudiantes amenazan y/o intimidan a los/as profesores/as.
X14	VEP04	Hay estudiantes que golpean a los/as profesores/as.
X15	VEP05	Algunos estudiantes rompen y/o roban pertenencias de los/as profesores/as.
X16	GCE01	El colegio propicia instancias de reflexión ante situaciones de violencia entre estudiantes.
X17	GCE02	El colegio realiza actividades para prevenir la violencia entre estudiantes, tales como: charlas, talleres, campañas.
X18	GCE03	En este colegio se aplican con claridad las sanciones cuando hay violencia entre estudiantes.
X19	GCE04	En este colegio existen personas capacitadas para ayudar a los estudiantes que se involucran en situaciones de violencia, ya sean víctimas, agresores o testigos.
X20	GCE07	Ante situaciones de violencia escolar, los estudiantes informan y/o piden ayuda con confianza a los adultos del establecimiento educacional.
X21	GCE09	Frente a situaciones de violencia entre estudiantes, los/as profesores/as recibimos apoyo oportuno del Encargado/a o Equipo de Convivencia Escolar.

Flavio Muñoz-Troncoso, Amy Halberstadt, Isabel Cuadrado-Gordillo, Enrique Riquelme-Mella, Edgardo Miranda-Zapata, Ekaterina Legaz-Vadímisrkaya, Valeria Sepúlveda-Bernales, Claudia Salamanca-Aroca, Gerardo Muñoz-Troncoso. 19-02-24
